# O‐GlcNAc‐induced nuclear translocation of hnRNP‐K is associated with progression and metastasis of cholangiocarcinoma

**DOI:** 10.1002/1878-0261.12406

**Published:** 2019-01-10

**Authors:** Chatchai Phoomak, Dayoung Park, Atit Silsirivanit, Kanlayanee Sawanyawisuth, Kulthida Vaeteewoottacharn, Marutpong Detarya, Chaisiri Wongkham, Carlito B. Lebrilla, Sopit Wongkham

**Affiliations:** ^1^ Department of Biochemistry Faculty of Medicine Khon Kaen University Thailand; ^2^ Cholangiocarcinoma Research Institute Khon Kaen University Thailand; ^3^ Department of Surgery Beth Israel Deaconess Medical Center Harvard Medical School Boston MA USA; ^4^ Department of Chemistry University of California Davis CA USA; ^5^ Center for Translational Medicine Faculty of Medicine Khon Kaen University Thailand

**Keywords:** bile duct cancer, heterogeneous nuclear ribonucleoprotein‐K, metastasis, O‐GlcNAcylated proteins

## Abstract

O‐GlcNAcylation is a key post‐translational modification that modifies the functions of proteins. Associations between O‐GlcNAcylation, shorter survival of cholangiocarcinoma (CCA) patients, and increased migration/invasion of CCA cell lines have been reported. However, the specific O‐GlcNAcylated proteins (OGPs) that participate in promotion of CCA progression are poorly understood. OGPs were isolated from human CCA cell lines, KKU‐213 and KKU‐214, using a click chemistry‐based enzymatic labeling system, identified using LC‐MS/MS, and searched against an OGP database. From the proteomic analysis, a total of 21 OGPs related to cancer progression were identified, of which 12 have not been previously reported. Among these, hnRNP‐K, a multifaceted RNA‐ and DNA‐binding protein known as a pre‐mRNA‐binding protein, was one of the most abundantly expressed, suggesting its involvement in CCA progression. O‐GlcNAcylation of hnRNP‐K was further verified by anti‐OGP/anti‐hnRNP‐K immunoprecipitations and sWGA pull‐down assays. The perpetuation of CCA by hnRNP‐K was evaluated using siRNA, which revealed modulation of cyclin D1, XIAP, EMT markers, and MMP2 and MMP7 expression. In native CCA cells, hnRNP‐K was primarily localized in the nucleus; however, when O‐GlcNAcylation was suppressed, hnRNP‐K was retained in the cytoplasm. These data signify an association between nuclear accumulation of hnRNP‐K and the migratory capabilities of CCA cells. In human CCA tissues, expression of nuclear hnRNP‐K was positively correlated with high O‐GlcNAcylation levels, metastatic stage, and shorter survival of CCA patients. This study demonstrates the significance of O‐GlcNAcylation on the nuclear translocation of hnRNP‐K and its impact on the progression of CCA.

**Abbreviations**
CCA: cholangiocarcinoma
EMT: epithelial to mesenchymal transition
GlcNAc: N‐acetylglucosamine
hnRNP‐K: heterogeneous nuclear ribonucleoprotein‐K
IHC: immunohistochemistry
MMP: matrix metalloproteinase
OGA: O‐GlcNAcase
OGPs: O‐GlcNAcylated proteins
OGT: O‐GlcNAc transferase
sWGA: succinylated wheat germ agglutinin

## 1 Introduction

O‐GlcNAcylation is a post‐translational modification of proteins in which a single sugar, N‐acetylglucosamine (GlcNAc), is covalently attached to the hydroxyl group of a serine or threonine residue on a polypeptide. The bioassembly is a dynamic process catalyzed by two enzymes, O‐GlcNAc transferase (OGT) and O‐GlcNAcase (OGA), which adds and removes the GlcNAc to and from the protein, respectively (Hart *et al*., 2007). Protein properties and functions are known to be modulated via O‐GlcNAcylation, for example, phosphorylation, interactions, degradation, and localization. Several evidences have indicated the association of aberrant O‐GlcNAcylation with many human diseases including cancer (Hart *et al*., 2011; Singh *et al*., 2015; Zachara and Hart, 2006). The significance of O‐GlcNAcylation in cancer metastasis has been demonstrated *in vitro* and *in vivo*. Suppression of OGT using shRNA resulted in inhibition of metastasis in xenografted mouse models of breast cancer (Ferrer *et al*., 2017; Gu *et al*., 2010), cervical cancer (Ali *et al*., 2017), and prostate cancer (Lynch *et al*., 2012).

We have previously reported the correlation of high O‐GlcNAcylation levels with shorter survival of cholangiocarcinoma (CCA) patients (Phoomak *et al*., 2012). Specifically, increased O‐GlcNAcylation of vimentin, a major intermediate filament protein, persuaded its stability and is implicated in the aggression of CCA cells. In addition, promotion of CCA aggressiveness under high glucose conditions was shown to be via elevation of OGT and O‐GlcNAcylation (Phoomak *et al*., 2017). On the other hand, suppression of OGT with siRNA significantly reduced cell migration and invasion of CCA cells (Phoomak *et al*., 2016). According to the O‐GlcNAcylated proteins database (dbOGAP) (Wang *et al*., 2011), there are only about 800 O‐GlcNAcylated proteins reported at present. In this context, there may be a number of O‐GlcNAcylated proteins (OGPs) associated with progression of cancer that remain unidentified. Historically, progress has been hampered in part by the technical difficulties in detection of OGPs (Hart *et al*., 2007). However, with the recent development of more sophisticated mass spectrometric methods in combination with biochemical tools, including enhancement of OGPs using OGA inhibitors, identification of OGPs has been markedly improved (Hart *et al*., 2007).

This study was aimed to determine novel OGPs that modulate progression of CCA cells. OGPs were first globally enriched and labeled using Click‐iT™ *O*‐GlcNAc Enzymatic Labeling System, and then identified using Q Exactive Plus Orbitrap mass spectrometry. Heterogeneous nuclear ribonucleoprotein‐K (hnRNP‐K) was selected and validated for its O‐GlcNAcylation status and involvement in CCA progression. The signal pathways related to hnRNP‐K in association with migration and invasion activities of CCA cells were subsequently determined. Specifically, O‐GlcNAcylation of hnRNP‐K was implicated in mediation of nuclear translocation in addition to migration of CCA cells. Moreover, association of O‐GlcNAcylation levels and hnRNP‐K expression was observed in tumor tissues of CCA patients in association with metastatic stage and shorter survival of patients. Significantly, these results implicate hnRNP‐K O‐GlcNAcylation as a promising therapeutic target to suppress CCA progression.

## 2 Materials and methods

### 2.1 Antibodies and reagents

Antibodies were purchased from various sources: anti‐O‐GlcNAc (RL‐2, MA1‐072) from Pierce Biotechnology (Rockford, IL, USA); anti‐hnRNP‐K (H‐300, sc‐25373), anticyclin D1 (H‐295, sc‐753), anti‐XIAP (H‐202, sc‐11426), anti‐MMP2 (H‐76, sc‐10736), anti‐MMP7 (JL07, sc‐80205), and anti‐OGT (F‐12, sc‐74546) from Santa Cruz Biotechnology (Santa Cruz, CA, USA); anticleaved caspase 3 (D175, 5A1E, #9664), anti‐E‐cadherin (24E10, #3195), anticlaudin‐1 (D5H1D, #13255), antivimentin (D21H3, #5741), and antislug (C19G7, #9585) from Cell Signaling (Danvers, MA, USA); PUGNAc (O‐(2‐acetamido‐2‐deoxy‐d‐glucopyranosylidene) amino‐N‐phenylcarbamate) from Sigma‐Aldrich (St. Louis, MO, USA).

### 2.2 CCA cell culture and CCA tissues

CCA cell lines (KKU‐100, KKU‐213, and KKU‐214) were obtained from the Japanese Collection of Research Bioresources (JCBR) Cell Bank (Osaka, Japan). MMNK1, an immortal cholangiocyte cell line, was a gift from Kobayashi N. (Maruyama *et al*., 2004). Cells were cultured in DMEM—Dulbecco's modified Eagle's medium (DMEM) (Gibco, Grand Island, NY, USA) supplemented with 10% FBS and 1% antibiotic–antimycotic under standard protocol. Transient enhancement of O‐GlcNAcylation was performed by culturing cells in the presence of 20 μm PUGNAc for 24 h prior to further experiments.

The immunohistochemistry (IHC) experiments were performed using formalin‐fixed paraffin‐embed liver tissues from histologically proven CCA patients. Each subject gave informed consent, and the study protocol was certified by the Ethics Committee for Human Research at Khon Kaen University (HE581369).

### 2.3 Identification of O‐GlcNAcylated proteins

The Click‐iT™ O‐GlcNAc Enzymatic Labeling System (Invitrogen, Carlsbad, CA, USA) was used to detect the OGPs in CCA cells. As shown in Fig. S1, cells were homogenized and N‐linked glycans were released as described previously (Park *et al*., 2016). Protein (2 mg) was trypsinized with 1 μg trypsin at 37°C overnight. The peptides were enriched with C‐18 column (Discovery^®^ DSC‐18, 52603U, Sigma) as standard protocol for solid‐phase extraction (Yang *et al*., 2016). O‐GlcNAcylated peptides were enzymatically labeled with azido‐modified galactose (GalNAz) by mutant β‐1,4‐galactosyltransferase (Gal‐T1 (Y289L)). The labeled peptides were tagged with biotin‐alkyne by Click‐iT™ Biotin Protein Analysis Detection Kit (Invitrogen). The complex was then pulled down with streptavidin–agarose resin (Thermo Scientific, Waltham, MA, USA) at 4 °C overnight. The peptides were cleaved by mild β‐elimination and Michael addition (BEMAD; 1.5% triethylamine, 20 mM dithiothreitol, pH 12‐12.5 with NaOH). The reaction was incubated at 54 °C for 4 h with shaking and stopped by addition of 2% trifluoroacetic acid. The peptides were enriched and analyzed using a Q Exactive Plus Orbitrap mass spectrometer (Thermo Scientific; Park *et al*., 2015). A 60‐min binary gradient was applied using 0.1% (v/v) formic acid in (A) water and (B) acetonitrile. The parameters of protein identification were set as follows: spray voltage 2.2 kV; ion transfer capillary temperature 200 °C; MS automatic gain control 1 × 10^6^; MS maximum injection time 30 ms; MS/MS automatic gain control 5 × 10^4^; MS/MS maximum injection time 50 ms; isolation width 1.6; normalized collision energy 27; charge state preference 2–8. The proteomics data were analyzed by X!Tandem (Craig and Beavis, 2004). Identified proteins were matched to the human proteome (SWISSPROT) and the Database of O‐GlcNAcylated Proteins and Sites (dbOGAP) (Wang *et al*., 2011).

### 2.4 Transient suppression of hnRNP‐K expression using specific siRNA

hnRNP‐K expression in CCA cells was suppressed using siRNA (Zhang *et al*., 2016b) as previously reported (Phoomak *et al*., 2016). Cells treated with scramble siRNA (Negative Control siRNA, 1027310, Qiagen, Hilden, Germany) were used as the control.

### 2.5 Cell proliferation

Viable cells were measured using the WST‐8 proliferation assay (Cell Counting Kit‐8 (CCK‐8), Dojindo Molecular Technologies, Inc., Rockville, MD, USA) according to the manufacturer's guidelines. The absorbance of soluble WST‐8 formazan was measured at 450 nm. Cell numbers were calculated as % of control cells.

### 2.6 Cell migration and invasion

CCA cells (40 000 cells) were placed into the upper chamber of a 8.0 μm pore size transwell‐cell culture inserts (Corning Incorporated, Corning, NY, USA) for migration and invasion assays as previously described (Phoomak *et al*., 2016). Cells were allowed to migrate or invade: 9 h for KKU‐213 and 24 h for KKU‐214. The migrated and invaded cells underneath the filter were stained and counted under a microscope with 10× objective lens. Experiments were performed in triplicate, and cells from 5 microscopic fields/insert were determined and calculated as % of control.

### 2.7 Immunoprecipitation

Cell lysate was prepared and immunoprecipitation was performed as previously described (Phoomak *et al*., 2016). Briefly, cell lysates (500 μg) were immunoprecipitated with 2 μg anti‐O‐GlcNAc or anti‐hnRNP‐K at 4 °C, overnight. The immunoprecipitated complex was separated and solubilized in SDS sample buffer prior to SDS/PAGE and western blotting.

### 2.8 Succinylated wheat germ agglutinin (sWGA) pull‐down assay

The sWGA pull‐down assay was performed as previously described (Kang *et al*., 2009). In brief, 500 μg of cell lysates was incubated with 40 μL of agarose‐conjugated sWGA (Vector Laboratories, Burlingame, CA, USA) with or without 0.25 m GlcNAc at 4 °C, overnight. The precipitates were washed four times with NET lysis buffer and boiled in SDS sample buffer.

### 2.9 SDS/PAGE and western blot analysis

Cells were lysed in lysis buffer (1% NP‐40, 150 mm NaCl, 50 mm Tris/HCl pH 7.4) containing 5 μm PUGNAc, phosphatase, and protease inhibitors. The SDS/PAGE and western blot were performed as previously described (Phoomak *et al*., 2016). The ECL™ Prime Western Blotting Detection System and the images were analyzed using an ImageQuant LAS 4000 mini image analyzer and ImageQuant™ TL analysis software (GE Healthcare, Buckinghamshire, UK).

### 2.10 Immunocytofluorescence

Cells were prepared for immunocytofluorescence as previously described (Phoomak *et al*., 2016). After fixation, cells were then incubated with 1:100 anti‐hnRNP‐K overnight at 4 °C and with 1:200 anti‐rabbit‐IgG‐PE (Santa Cruz) for 1 h at room temperature. To visualize nuclei, cells were stained with 1:10 000 Hoechst 33342 (Molecular Probes, Invitrogen, Paisley, UK). The fluorescence image was taken using a ZEISS LSM 800 Confocal Laser Scanning Microscope (Zeiss, Oberkochen, Germany).

### 2.11 Immunohistochemistry

Expression of hnRNP‐K and OGP in CCA tissues was determined using immunohistochemistry (IHC) staining according to the standard protocol. The signals were amplified using the EnVision‐system‐HRP (Dako, Glostrup, Denmark). The immunoreactivity signals were developed using diaminobenzidine (Sigma‐Aldrich). The IHC score was determined as described previously (Phoomak *et al*., 2017). Two independent assessors scored the levels of IHC staining signal blindly without prior knowledge of clinical parameters.

### 2.12 Statistical analysis

All statistics were analyzed using the GraphPad Prism^®^ 5.0 software (GraphPad software, Inc., La Jolla, CA, USA). Student's *t*‐test was used to compare parameters between two samples. The correlation between OGP level and hnRNP‐K expression in CCA patient tissues was determined using Fisher's exact test, Mann–Whitney test, and Spearman's rank correlation test. Differences were considered statistically significant if *P *<* *0.05.

## 3 Results

### 3.1 Increasing O‐GlcNAcylation enhances migration and invasion abilities of CCA cells

**Figure 1:**
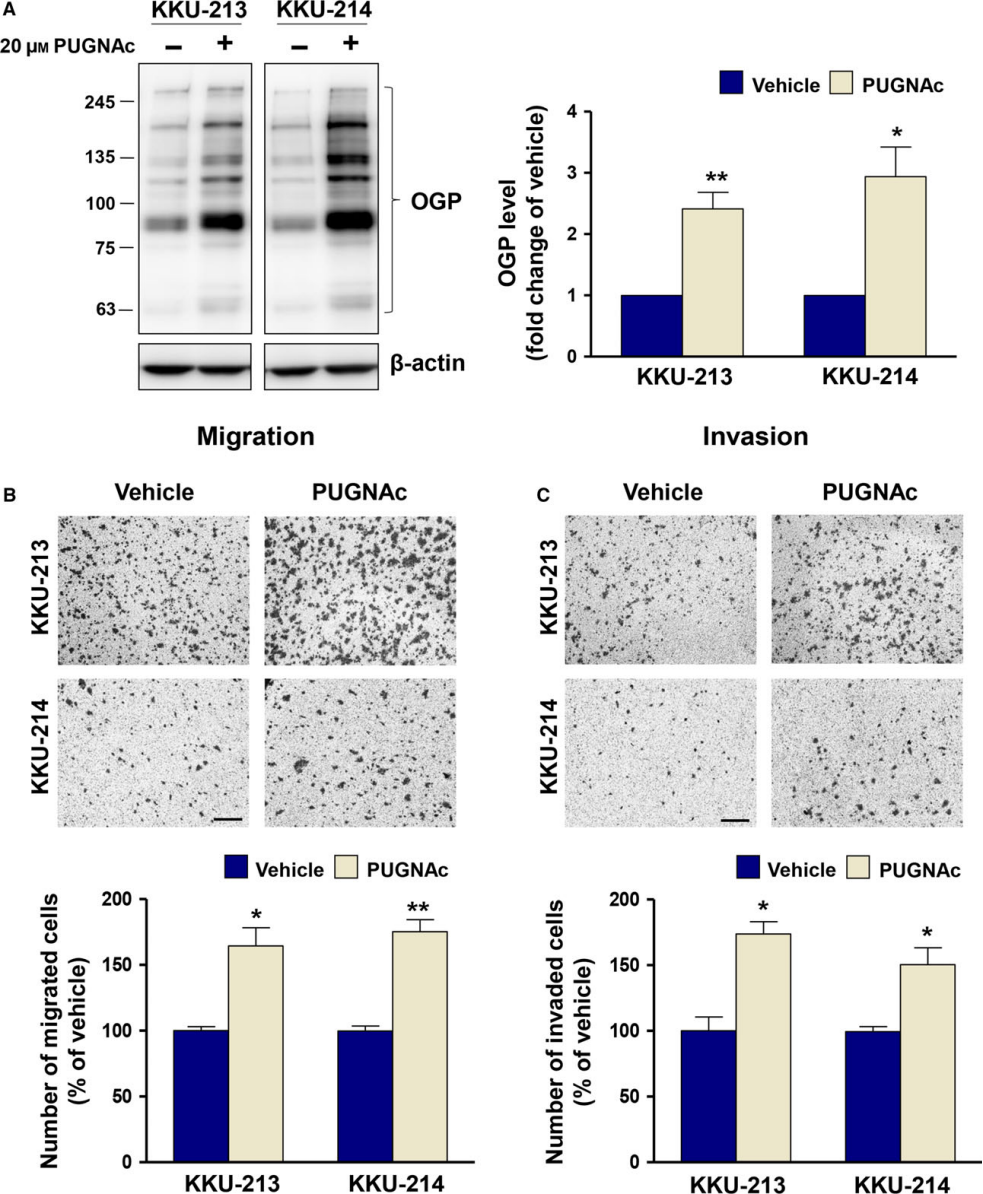
O‐GlcNAcylation promotes CCA migration and invasion. CCA cells, KKU‐213 and KKU‐214, were treated with 20 μm PUGNAc for 24 h. (A) OGP levels were determined using western blot. (B) Migration and (C) invasion abilities of PUGNAc‐treated CCA cells were compared with those of the vehicle control cells. The results represent one of two independent experiments (mean ± SEM, *P < 0.05; **P < 0.01, Students’ *t*‐test). The images shown are 100 ×  magnification with 50 μm of scale bar.

As PUGNAc, an inhibitor of OGA was used to enrich the O‐GlcNAcylation in CCA cells, we first determined whether PUGNAc treatment could increase O‐GlcNAcylation and enhance progression of CCA cells. CCA cells (KKU‐213 and KKU‐214) were treated with PUGNAc for 24 h, and the OGP level together with migration and invasion abilities of CCA cells treated with or without PUGNAc was determined. As shown in Fig. 1A, suppression of OGA activity using PUGNAc increased the levels of OGP in CCA cells 2.5‐fold in KKU‐213 and 3.0‐fold in KKU‐214, respectively. PUGNAc treatment also significantly enhanced the relative migratory ability to 165% in KKU‐213 and to 175% in KKU‐214 compared with the control cells (Fig. 1B). Similar results were also observed for the invasion ability. PUGNAc treatment increased invasion of KKU‐213 to 175% and of KKU‐214 to 150% compared with those of control cells (Fig. 1C).

### 3.2 Novel O‐GlcNAcylated proteins related to progression of CCA cells were revealed by enzymatic labeling and mass spectrometry analysis

To increase the sensitivity of OGP detection, O‐GlcNAcylated peptides were labeled with GalNAz by GalT1 (Y289L) and tagged with biotin‐alkyne. The labeled peptides were then analyzed by mass spectrometry (Fig. S1). Over 100 OGPs were identified in cell lysates from KKU‐213 and KKU‐214 (Tables S1), of which the major OGPs were found in the cytoplasm and nucleus (Fig. S2A).

To classify the OGPs that are related to progression of CCA cells, the primary list of OGPs obtained from mass spectrometry were filtered according to the following parameters: (1) it was present in both KKU‐213 and KKU‐214 cells, and (2) it had at least one predicted O‐GlcNAcylation site (based on dbOGAP). There were 21 OGPs that passed these criteria. The description, cellular localization, and functions of these OGPs are listed according to the intensity of the peptides in Table 1. Twelve OGPs listed may be novel OGPs as their O‐GlcNAcylation has not been identified (Fig. S2B). The involvement of these OGPs in biological processes is summarized in Fig. S2C.

**Table 1:** List of O‐GlcNAcylated proteins related with proliferation and progression of cancer

UniProt accession	Gene name	Protein description	log(intensity)^a^	Cellular compartment^b^	Function	Reference
Reported O‐GlcNAcylated proteins						
P60709	ACTB	Actin, beta	8.33	Cytoplasm	–	–
P16403	HIST1H1C	Histone cluster 1 H1c	7.78	Nucleus	Proliferation	Song *et al*. (2008)
					Migration	
					Invasion	
P10412	HIST1H1E	Histone cluster 1 H1e	7.74	Nucleus	Proliferation	Lee *et al*. (2013)
P16401	HIST1H1B	Histone cluster 1 H1b	7.69	Nucleus	Carcinogenesis	Khachaturov *et al*. (2014)
P02545	LMNA	Lamin A/C	7.56	Nucleus	Proliferation	Kong *et al*. (2012)
					Migration	
					Invasion	
P06748	NPM1	Nucleophosmin (nucleolar phosphoprotein B23 numatrin)	7.49	Cytoplasm	Proliferation	Ching *et al*. (2015)
				Nucleus	Migration	
					Invasion	
P22626	NRNPA2B1	Heterogeneous nuclear ribonucleoprotein A2/B1	7.32	Nucleus	Proliferation	Chen *et al*. (2014)
					Migration	
					Invasion	
Q09666	AHNAK	AHNAK nucleoprotein	7.10	Nucleus	Proliferation	Sudo *et al*. (2014)
					Migration	
					Invasion	
P07355	ANXA2	Annexin A2	7.07	Membrane	Proliferation	Chaudhary *et al*. (2014) and Wang *et al*. (2015)
				Cytoplasm	Migration	
				Nucleus	Invasion	
Unreported O‐GlcNAcylated proteins						
P46939	UTRN	Utrophin	8.49	Membrane	Proliferation	Li *et al*. (2007)
				Cytoplasm		
Q5QNW6	HIST2H2BF	Histone cluster 2 H2bf	8.43	Nucleus	–	–
P62805	HIST1H4A	Histone cluster 2 H4a	8.16	Nucleus	Proliferation	Yan‐Fang *et al*. (2015)
B9ZVM9	TCP10L2	T‐complex protein 10A homolog 2	7.95	Nucleus	Proliferation	(Shen *et al*., 2015)
Q16695	HIST3H3	Histone cluster 3 H3	7.50	Nucleus	Proliferation	(Xu *et al*., 2014)
P08195	SLC3A2	Solute carrier family 3 (amino acid transporter heavy chain), member 2	7.09	Membrane	Proliferation	Fei *et al*. (2014), Santiago‐Gomez *et al*. (2013) and Yang *et al*. (2007)
				Cytoplasm	Migration	
				Nucleus	Invasion	
Q16819	MEP1A	Meprin A subunit alpha	6.96	Membrane	Migration	Minder *et al*. (2012)
					Invasion	
P61978	HNRNPK	Heterogeneous nuclear ribonucleoprotein‐K	6.81	Cytoplasm	Proliferation	Chung *et al*. (2014) and Gao *et al*. (2013)
				Nucleus	Migration	
					Invasion	
P27824	CANX	Calnexin	6.73	Cytoplasm	Carcinogenesis	Dissemond *et al*. (2004)
					Metastasis	
Q08170	SRSF4	Serine/arginine‐rich splicing factor 4	6.26	Nucleus	Proliferation	Gabriel *et al*. (2015)
Q5T200	ZC3H13	Zinc finger CCCH‐type containing 13			–	–
Q7Z7G8	VPS13B	Vacuolar protein sorting 13 homolog B			–	–

a Log (intensity) of identified OGPs in KKU‐213.

b According to GeneCards^®^: The Human Gene Database.

### 3.3 Immunoprecipitation reveals O‐GlcNAc modification of hnRNP‐K

hnRNP‐K, a multifaceted RNA‐ and DNA‐binding protein associated with pre‐mRNA, mRNA metabolism and transport (Dejgaard and Leffers, 1996; Lu and Gao, 2016), has been shown to contribute to metastasis in several cancer types (Chung *et al*., 2014; Gao *et al*., 2013; Zhang *et al*., 2016b; Zhou *et al*., 2010). Moreover, hnRNP‐K possesses multiple Ser/Thr sites that are predicted to be O‐GlcNAcylated. Therefore, hnRNP‐K was selected for verification of its O‐GlcNAc modification and involvement in CCA progression.

**Figure 2:**
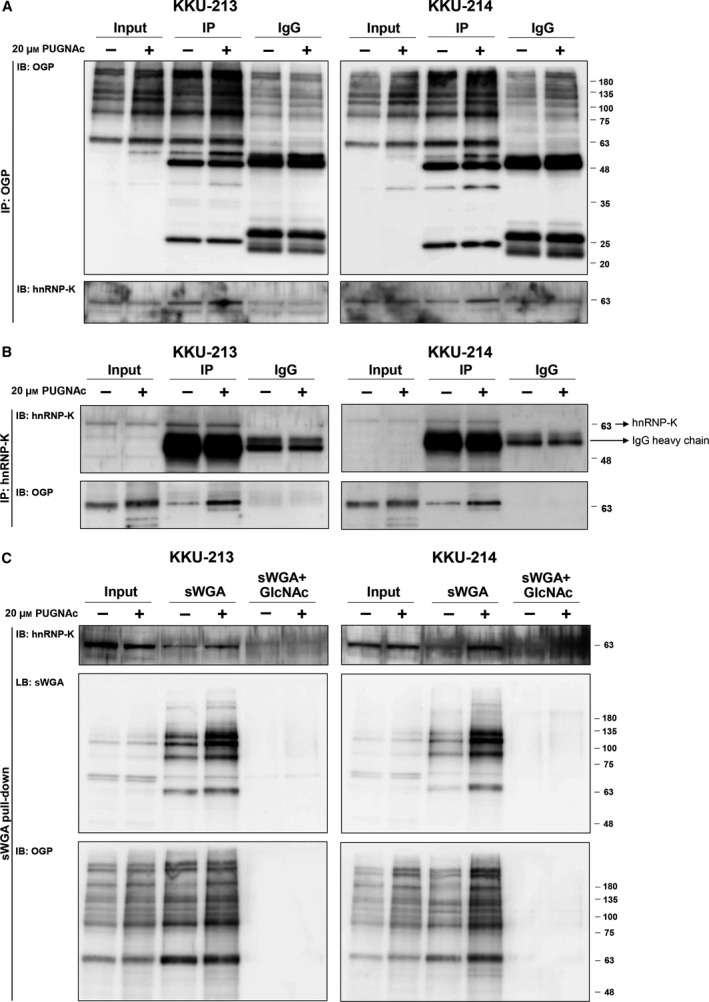
Validation of hnRNP‐K O‐GlcNAcylation. CCA cells (KKU‐213 and KKU‐214) were treated with PUGNAc or vehicle for 24 h. The cell lysates were immunoprecipitated with either (A) anti‐OGP or (B) anti‐hnRNP‐K and probed with anti‐hnRNP‐K and anti‐OGP. Human immunoglobulin G (IgG) isotype was used as the controls of the specificity of the antibodies that were used in the immunoprecipitation assay. (C) The sWGA pull‐down assay was performed using sWGA‐conjugated agarose and probed with anti‐hnRNP‐K, sWGA, and anti‐OGP. GlcNAc neutralization was used to examine the specific binding of sWGA to the OGPs.

To prove the modification of O‐GlcNAc on hnRNP‐K, an immunoprecipitation assay was performed. Cell lysates of CCA cells treated with PUGNAc or vehicle were subjected to immunoprecipitation using anti‐OGP. Immunoprecipitation using mouse immunoglobulin (IgG) as an isotype control was used to clarify the specificity of the anti‐OGP. As shown in Fig. 2A, PUGNAc treatment increased the expression level of OGPs and signal of hnRNP‐K in the immunoprecipitated‐OGP from both KKU‐213 and KKU‐214 cells. Similar results were obtained for the reversed‐immunoprecipitation using anti‐hnRNP‐K (Fig. 2B). In both cell lines, the signal of OGP was higher in the immunoprecipitate of hnRNP‐K from PUGNAc‐treated cells than that from the control cells. These data demonstrated the O‐GlcNAc modification of hnRNP‐K. As succinylated wheat germ agglutinin (sWGA) specifically recognizes the sugar moiety of GlcNAc, an sWGA pull‐down assay was performed to further ensure that hnRNP‐K was O‐GlcNAcylated. As shown in Fig. 2C, the signal of hnRNP‐K in the sWGA pull‐down precipitate from PUGNAc‐treated cells was higher than that of the control cells. The specific interaction between O‐GlcNAcylated hnRNP‐K and sWGA was assured by the neutralization of sWGA with GlcNAc. The signals of O‐GlcNAcylated hnRNP‐K, sWGA‐conjugated proteins, and O‐GlcNAcylated proteins were diminished in the GlcNAc‐neutralized sWGA condition. In addition, the level of O‐GlcNAcylation of hnRNP‐K was elevated when cellular O‐GlcNAcylation was increased. Collectively, these results indicate the O‐GlcNAcylation of hnRNP‐K.

### 3.4 hnRNP‐K is required for cell proliferation, migration, and invasion of CCA cells

**Figure 3:**
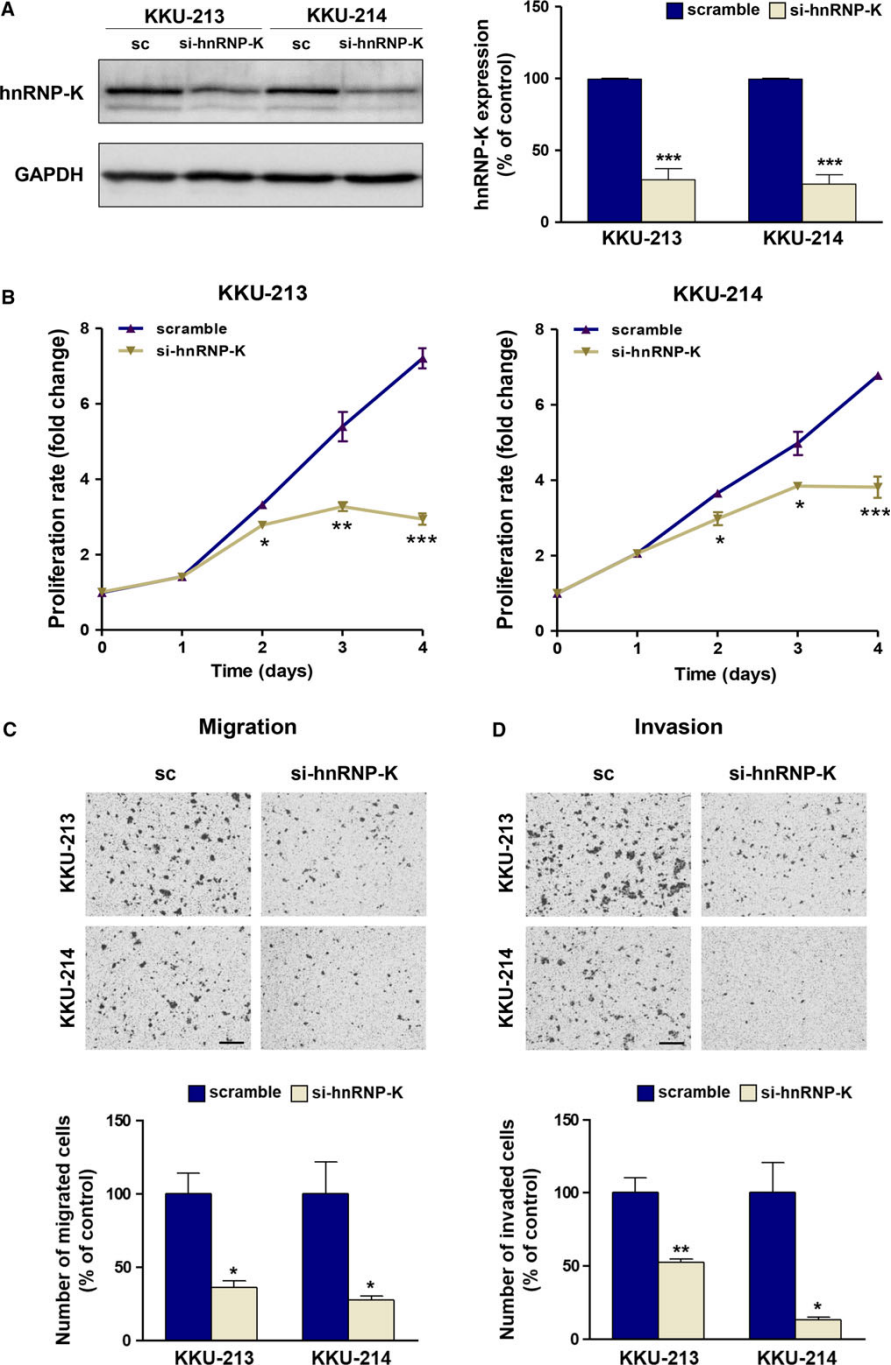
Suppression of hnRNP‐K reduces proliferation, migration, and invasion of CCA cells. The expression of hnRNP‐K was transiently suppressed by siRNA for 48 h prior to the migration and invasion assays. (A) The expression of hnRNP‐K was determined using western blot. (B) Cell proliferation, (C) migration, and (D) invasion abilities of si‐hnRNP‐K‐treated CCA cells were compared with those of the scramble siRNA (sc)‐treated cells. The migration and invasion assays were conducted for 9 h in KKU‐213 and 24 h in KKU‐214. The images shown are 100 ×  magnification with 50 μm scale bar. Data are mean ± SEM (*P < 0.05; **P < 0.01; ***P < 0.001, Students’ *t*‐test).

We next investigated the involvement of hnRNP‐K in CCA progression, indicated namely by increases in cell proliferation, migration, and invasion. To this end, the expression of hnRNP‐K was transiently suppressed by siRNA, and cell proliferation, migration, and invasion were determined in comparison with those of the scramble control cells. The si‐hnRNP‐K transfection could reduce the expression of hnRNP‐K to 30% of the control cells in KKU‐213 and to 25% in KKU‐214 (Fig. 3A). Proliferation rates of KKU‐213 and KKU‐214 were significantly decreased when the expression of hnRNP‐K was suppressed for 48 h (Fig. 3B). Moreover, diminution of hnRNP‐K expression markedly decreased the motility of CCA cells to 36% of the control cells in KKU‐213 and to 27% in KKU‐214 (Fig. 3C). Similar effects were also observed for the invasion ability of CCA cells. The invasion ability of si‐hnRNP‐K‐treated cells was 50% and 15% of the control cells in KKU‐213 and KKU‐214, respectively (Fig. 3D). These data indicated the association of hnRNP‐K with the proliferation, migration and invasion of CCA cells. To ensure that the observed effects of hnRNP‐K on migration and invasion were not due to changes in growth rates, we parallelly measured cell proliferation. As shown in Fig. S3, there were no growth differences between si‐hnRNP‐K‐ and scramble siRNA‐treated cells during the time of assays. Thus, si‐hnRNP‐K conferred decreases in migration and invasion without affecting cell growth.

### 3.5 Key markers related to growth and metastasis proteins are influenced by hnRNP‐K

**Figure 4:**
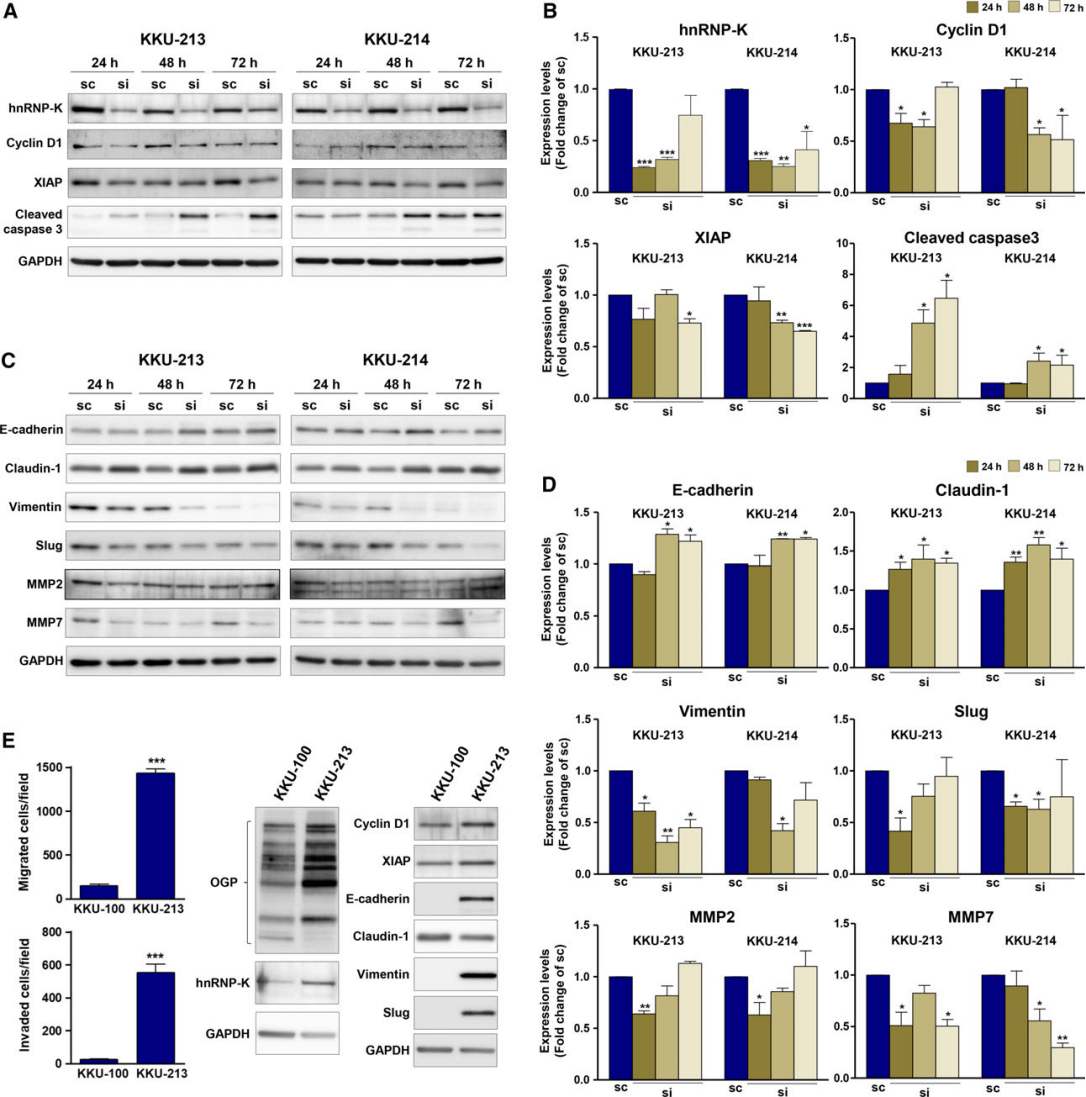
hnRNP‐K mediates the expression of growth‐ and metastasis‐related proteins in CCA cells. CCA cell lines, KKU‐213 and KKU‐214, were treated with si‐hnRNP‐K for 24, 48, and 72 h. The expression levels of growth‐ and metastasis‐related proteins were determined using western blot. Expression of GAPDH was used as an internal control. The expression of (A) hnRNP‐K, cyclin D1, XIAP, and cleaved caspase 3 as well as (C) EMT markers (E‐cadherin, claudin‐1, vimentin, and slug), and MMP2 and MMP7 was determined in si‐hnRNP‐K‐treated cells in comparison with those of scramble control cells. B and D are the quantitative analysis of (A) and (C) presented as mean ± SD from two independent experiments. (E) The endogenous expression of hnRNP‐K and its downstream targets was compared in 2 CCA cell lines (KKU‐100 and KKU‐213) with different migration/invasion abilities and O‐GlcNAcylation levels. (*P < 0.05; **P < 0.01, Students’ *t*‐test)

Given that hnRNP‐K is a multifaceted RNA‐ and DNA‐binding protein, we further examined the influence of hnRNP‐K on key effector proteins related to these malignant phenotypes: cyclin D1 for cell proliferation, XIAP for antiapoptosis, cleaved caspase 3 for cell apoptosis, E‐cadherin and claudin‐1 for epithelial markers, vimentin and slug for mesenchymal markers, and MMP2 and MMP7 for invasion activity. Specifically, the expression of cyclin D1 and XIAP was investigated after hnRNP‐K was suppressed by siRNA for 24, 48, and 72 h. Compared to the control cells, the expression of cyclin D1 and XIAP in si‐hnRNP‐K‐treated KKU‐213 and si‐hnRNP‐K‐treated‐KKU‐214 cells decreased along with hnRNP‐K expression until 72 h (Fig. 4A). In addition, the level of cleaved caspase 3 increased with time in si‐hnRNP‐K‐treated cells. The quantitative data are shown in Fig. 4B.

To determine the effect of hnRNP‐K on the effector proteins related to cell migration and invasion, the expression of epithelial to mesenchymal transition (EMT) markers (e.g., E‐cadherin, claudin‐1, vimentin, and slug), and matrix metalloproteinase (MMP) 2 and MMP7 was determined in si‐hnRNP‐K‐treated cells in comparison with those of the scramble control cells. As shown in Fig. 4C,D, the expression levels of E‐cadherin and claudin‐1 increased whereas those of vimentin and slug decreased in si‐hnRNP‐K‐treated cells. On the other hand, while si‐hnRNP‐K treatment suppressed the MMP2 expression after 24 h of treatment, expression of MMP7 gradually decreased with time. These data demonstrated that hnRNP‐K influenced cell migration and invasion in association with the expression of EMT, MMP2, and MMP7.

To emphasize the connection of O‐GlcNAcylation levels, hnRNP‐K and its downstream signals, the expression level of O‐GlcNAcylation, hnRNP‐K, cyclin D1, XIAP, and EMT markers was determined in CCA cell lines, KKU‐100, which shows lower migration activity versus KKU‐213. As shown in Fig. 4E, compared to KKU‐213, KKU‐100 exhibited not only lower levels of O‐GlcNAcylation but also lower levels of hnRNP‐K and the effector molecules related to migration and invasion.

To investigate whether enhancing O‐GlcNAcylation of hnRNP‐K could support the migratory activity of cells, two additional cell lines with low hnRNP‐K expression were enrolled, MMNK1, an immortal cholangiocyte, and KKU‐100. Their migration was measured with si‐hnRNP‐K treatment and in the presence or absence of PUGNAc. While siRNA of hnRNP‐K was used to suppress the expression of hnRNP‐K, PUGNAc treatment enhanced the levels of OGPs in both cell lines (Fig. S4). Suppression of hnRNP‐K expression decreased the migratory activity of MMNK1 and KKU‐100 cells to 50% and 30% of the control cells, respectively. On the other hand, elevating O‐GlcNAcylation by PUGNAc treatment increased the migratory ability of both the control and si‐hnRNP‐K‐treated cells. Similar effects were observed in MMNK1 and KKU‐100. Together, these results support our finding that O‐GlcNAcylation and hnRNP‐K are associated with migratory ability regardless of cell type.

### 3.6 O‐GlcNAcylation of hnRNP‐K activates the nuclear translocation of hnRNP‐K

**Figure 5:**
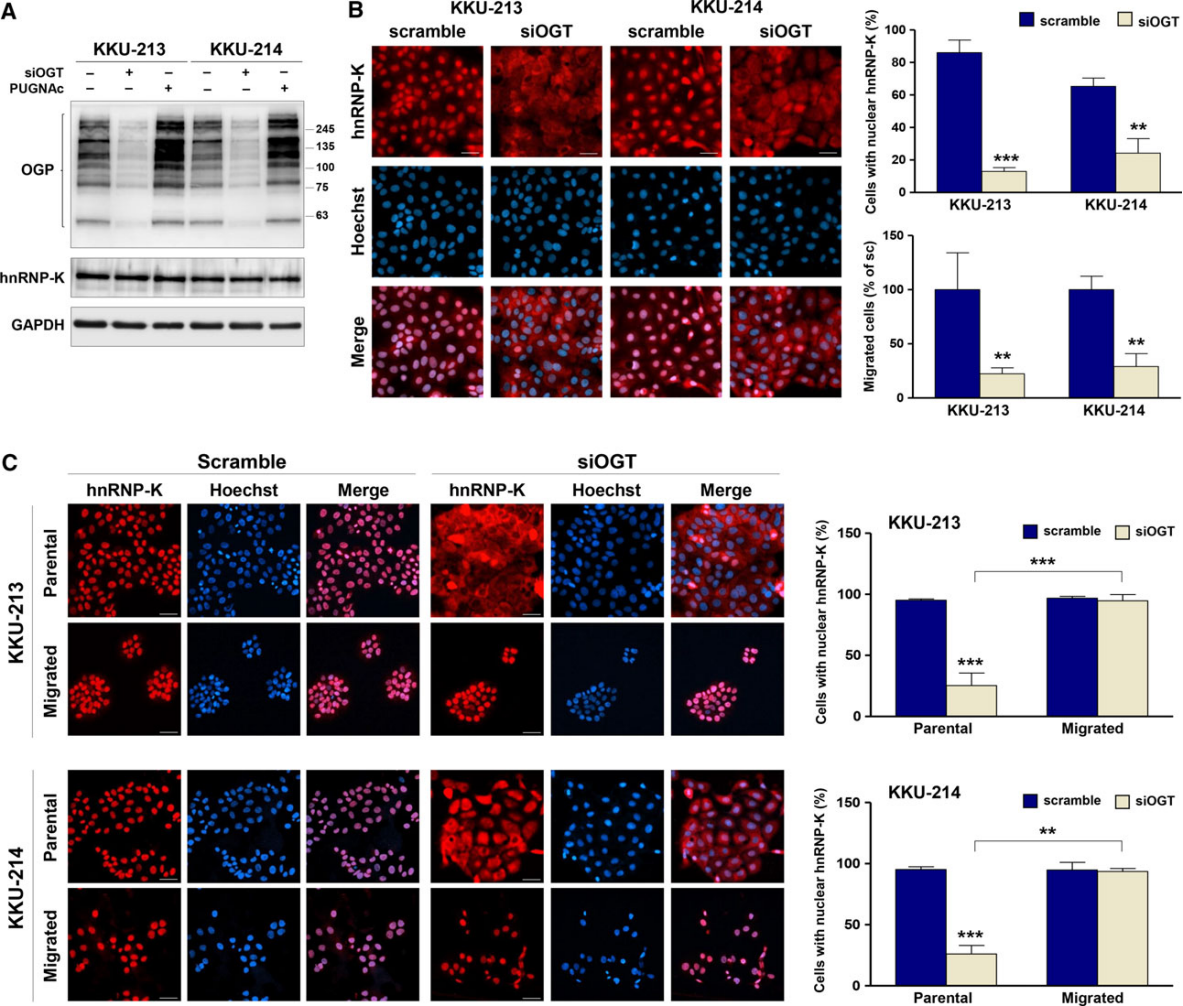
hnRNP‐K nuclear localization is regulated by O‐GlcNAcylation and correlated with migratory activity of CCA cells. (A) The expression of OGT was suppressed by siRNA for 24 h. Expression levels of OGP and hnRNP‐K in siOGT‐ and PUGNAc‐treated cells were compared with those of control cells using western blot analysis. (B) Localization of hnRNP‐K and nuclei was observed using immunocytofluorescent staining. hnRNP‐K was stained using PE (red), and cell nuclei were visualized using Hoechst 33342 (blue). Nuclear localization of hnRNP‐K is demonstrated by the purple nuclei in the merged images. The quantification of scramble siRNA‐ and siOGT‐treated cells with nuclear hnRNP‐K is shown in the upper panel graph. The migratory ability of cells treated with scramble siRNA and siOGT is shown in the lower panel graph. (C) The scramble siRNA‐ and siOGT‐treated cells were allowed to migrate to the lower chamber in a Transwell culture system for 48 h. Localization of hnRNP‐K was determined in the parental and migrated cells using immunocytofluorescent staining. Cells with nuclear hnRNP‐K were counted as shown in the graphs. The images are 200 ×  magnification and scale bars = 20 μm. Data are mean ± SD with **P < 0.01; ***P < 0.001 (Students’ *t*‐test).

To investigate the effect of O‐GlcNAcylation on the function of hnRNP‐K, the level of O‐GlcNAcylation was monitored using siOGT and PUGNAc. As shown in Fig. 5A, siOGT treatment reduced cellular O‐GlcNAcylation whereas PUGNAc treatment increased the level of O‐GlcNAcylation in both KKU‐213 and KKU‐214. However, neither treatments affected hnRNP‐K expression. This observation implied that O‐GlcNAcylation may not affect the expression and stability of hnRNP‐K.

As hnRNP‐K is a transcription factor and the translocation from cytoplasm to nucleus is an important process for proper functioning of hnRNP‐K, we next explored the effect of O‐GlcNAcylation on the nuclear translocation of hnRNP‐K. Cellular localization of hnRNP‐K was determined in siOGT‐treated cells using hnRNP‐K immunocytofluorescence: hnRNP‐K was stained using PE (red) and cell nuclei were visualized using Hoechst 33342 (blue). As shown in Fig. 5B, almost all of the positive hnRNP‐K signals of scramble control cells were located in the nucleus (red nuclei of the hnRNP‐K staining; purple nuclei of the merged images). Suppression of O‐GlcNAcylation in siOGT‐treated cells retained hnRNP‐K signals in the cytoplasm (red cytoplasmic stain with blue nuclei of the merged images). The number of cells with positive nuclear hnRNP‐K was significantly reduced in siOGT‐treated cells in both KKU‐213 (*P *<* *0.001) and KKU‐214 (*P *<* *0.01; Fig. 5B). The siOGT treatment also significantly decreased migratory activity of both cell lines. These data suggested that O‐GlcNAcylation modulates the nuclear localization of hnRNP‐K, which may in turn influence the migratory ability of CCA cells.

To affirm the connection between O‐GlcNAcylation and nuclear localization of hnRNP‐K, the expression and localization of hnRNP‐K in KKU‐100 were coevaluated using immunofluorescent staining. As shown in Fig. S5A‐B, the number of KKU‐100 cells with nuclear hnRNP‐K was significantly lower than that of KKU‐213 and KKU‐214 (*P *<* *0.05). The difference corresponded with the level of O‐GlcNAcylation and hnRNP‐K expression. In addition, increased O‐GlcNAcylation by PUGNAc treatment in KKU‐100 resulted in a higher proportion of cells with nuclear hnRNP‐K (Fig. S5C). These data are consistent with those observed in KKU‐213 and KKU‐214 cells in that O‐GlcNAcylation conferred the nuclear translocation of hnRNP‐K.

### 3.7 Migratory enhancement of CCA cells is correlated with nuclear translocation of hnRNP‐K

To connect the nuclear translocation of hnRNP‐K with the migratory ability of CCA cells, the localization of hnRNP‐K in the parental and migrated cells was determined. CCA cells were transfected with scramble siRNA or siOGT for 24 h and allowed to migrate to the lower chamber of a Transwell system for 48 h. Localization of hnRNP‐K in the parental cells and the migrated cells in the lower chamber were detected using immunocytofluorescent staining. As shown in Fig. 5C, almost all the parental and migrated si‐scramble‐treated cells possessed nuclear hnRNP‐K (purple nuclei). Suppression of O‐GlcNAcylation using siOGT, however, resulted in the retention of hnRNP‐K in the cytoplasm (pink cytoplasm with blue nuclei) and significantly reduced the number of cells with nuclear hnRNP‐K (*P *<* *0.001). Furthermore, when siOGT‐treated cells were allowed to migrate to the lower compartment of the Boyden chamber, it was found that only the fraction of cells with nuclear hnRNP‐K could migrate to the lower chamber. These data emphasized the association of nuclear hnRNP‐K and migratory activity of CCA cells.

### 3.8 Expression of nuclear hnRNP‐K in CCA tissues positively correlates with O‐GlcNAcylation levels

**Figure 6:**
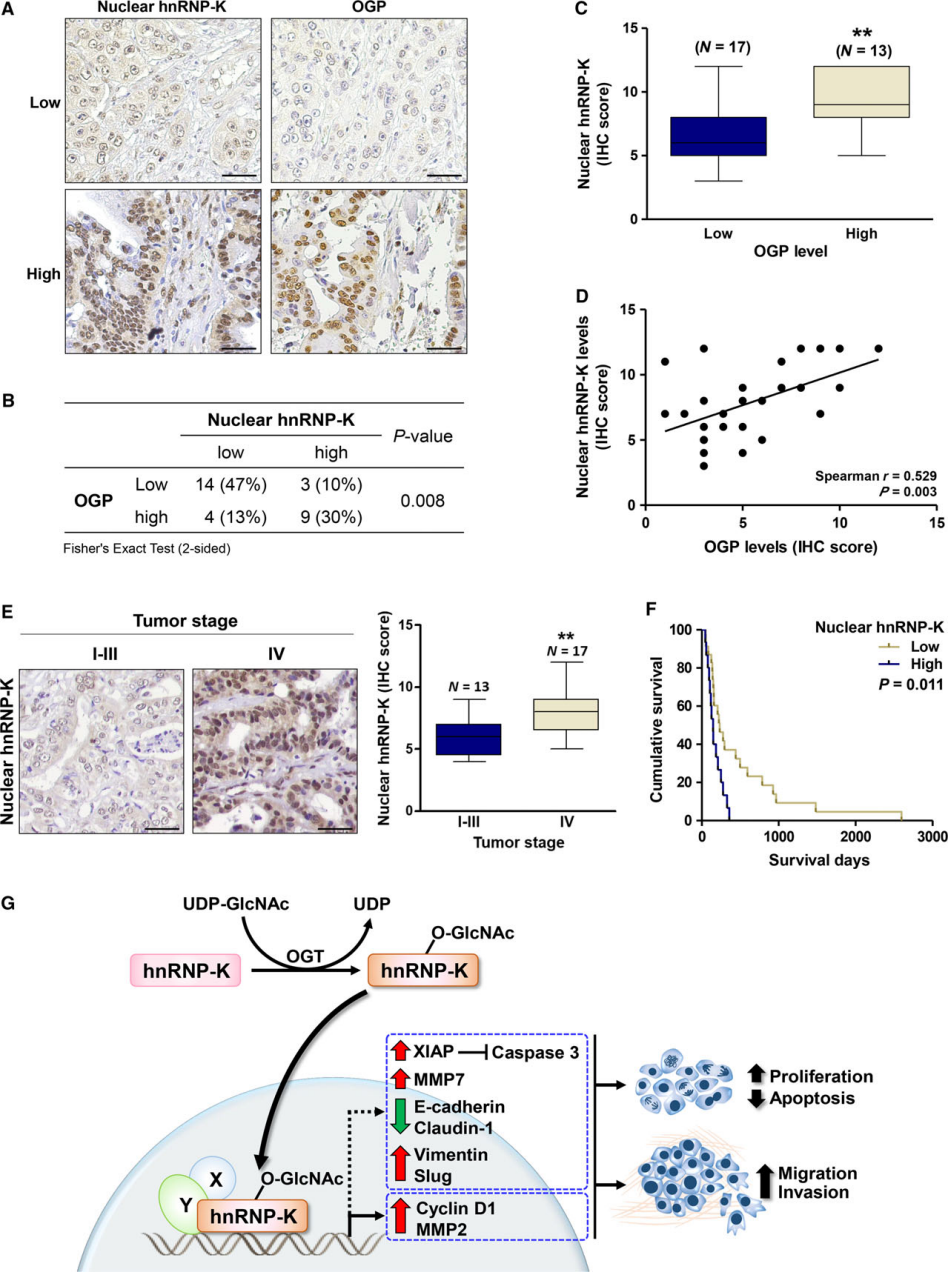
The expression and localization of hnRNP‐K in CCA tissues correlated with OGP levels. (A) IHC staining of hnRNP‐K and OGP in two representative pairs of CCA tissues. (B) The correlations between hnRNP‐K expression and OGP were analyzed by Fisher's exact test (*N* = 30). (C) Mean expression of hnRNP‐K in nucleus of the high OGP group was significantly higher than those of the low OGP group (**P < 0.01; Mann–Whitney test). (D) The positive correlation between nuclear hnRNP‐K and OGP levels was shown by Spearman's rank correlation test. (E) Nuclear hnRNP‐K in CCA patient tissues with stage I‐III and IV were compared. (F) High level of nuclear hnRNP‐K was correlated with shorter CCA patients with high nuclear hnRNP‐K level exhibited the shorter survival (median survival = 147 days, 95% CI = 106–187 days), than those with low nuclear hnRNP‐K level (median survival = 233 days, 95% CI = 128‐338 days) (*P* = 0.011, Kaplan–Meier plot and log‐rank test). The IHC images are showed in 200 ×  magnification with 20 μm of scale bar. (G) Schematic diagram presents molecular mechanism by which O‐GlcNAcylation regulated nuclear translocation of hnRNP‐K in association with progression of CCA.

Upon observation of O‐GlcNAc‐mediated nuclear translocation of hnRNP‐K in CCA cell lines, we then verified whether this association could be observed in tumor tissues of CCA patients. The expression levels of OGP and hnRNP‐K with nuclear localization were determined in 30 cases of CCA tissues using IHC, semiquantitated according to the intensity and frequency of the positive signal with IHC scores. hnRNP‐K and OGP were generally observed in both the nucleus and cytoplasm of CCA tissues but nuclear staining with different intensities was predominantly observed (Fig. 6A). Expression of nuclear hnRNP‐K and OGP was categorized according to the median of IHC scores as low or high levels, and the correlation of these two factors was analyzed. Positive correlations between number of CCA cells with nuclear hnRNP‐K and those with OGP expression were observed (Fig. 6B, Fisher's exact test). CCA tissues with high nuclear hnRNP‐K expression also had high OGP expression. In addition, higher expression of nuclear hnRNP‐K was observed in CCA tissues with high OGP expression than those with low OGP expression (Fig. 6C, Mann–Whitney test). Correlations between the expression levels of nuclear OGP and those of nuclear hnRNP‐K are shown by Spearman rank correlation with *r* = 0.529 (Fig. 6D).

### 3.9 High expression of tissue nuclear hnRNP‐K is associated with metastatic stage and poor clinical outcome of CCA patients

To implicate the clinical significance of hnRNP‐K in CCA, expression of tissue hnRNP‐K and clinicopathological features of CCA patients were determined in 38 CCA subjects. Nuclear hnRNP‐K expression was categorized as low or high based on median IHC score, and univariate analysis was performed. CCA tissues with metastatic stage (stage IV) exhibited higher levels of nuclear hnRNP‐K than those with nonmetastatic stages (stages I–III) (Table 2, Fig. 6E). The Kaplan–Meier analysis indicated that patients whose tumor possessed high nuclear hnRNP‐K had significantly shorter survival than those possessed low nuclear hnRNP‐K (Fig. 6F, *P* = 0.011, log‐rank analysis). Univariate Cox proportional hazard‐regression analysis was next performed to determine the influence of nuclear hnRNP‐K levels and clinicopathological characteristics on overall survival of CCA patients. As shown in Table 3, high level of nuclear hnRNP‐K was significantly correlated with overall survival (*P *=* *0.014) and an independent prognostic factor of CCA (HR = 2.540, 95% CI = 1.213–5.317, *P *=* *0.013).

**Table 2:** The correlation between nuclear hnRNP‐K levels and clinicopathological data of CCA patients

Variables (*N*)	Nuclear hnRNP‐K		*P*‐value
	Low	High	
Age (38)			
≤ 56 (21)	13	8	0.847
> 56 (17)	10	7	
Sex (38)			
Male (25)	16	9	0.544
Female (13)	7	6	
Histological type (38)			
Papillary (11)	7	4	0.802
Nonpapillary (27)	16	11	
CCA stage (30)			
I–III (13)	12	1	0.001*
IV (17)	5	12	

*P < 0.001, Fisher's exact test (two‐sided).

**Table 3:** Univariate and multivariate analysis of factors influencing overall survival in CCA patients

Variables (*N*)	Univariate analysis			Multivariate analysis		
	HR	95% CI	*P*‐value	HR	95% CI	*P*‐value
Age (38)						
≤ 56 (21)	1					
> 56 (17)	0.741	0.384‐1.428	0.370			
Sex (38)						
Male (25)	1					
Female (13)	1.302	0.650‐2.609	0.456			
Histological type (38)						
Papillary (11)	1			1		
Nonpapillary (27)	1.353	0.676‐2.706	0.393	1.439	0.702‐2.949	0.320
CCA stage (30)						
I–III (13)	1					
IV (17)	1.850	0.835‐4.102	0.130			
Nuclear hnRNP‐K (38)						
Low (23)	1			1		
High (15)	2.528	1.208‐5.294	0.014*	2.540	1.213‐5.317	0.013*

CI, confidence interval; HR, hazard ratio.

*P < 0.05, Cox proportional hazard‐regression test.

## 4 Discussion

Several O‐GlcNAcylated proteins (OGPs) have been reported for their roles in cancer proliferation, metastasis, metabolism, angiogenesis, stress response, replicative immortality, and resistance to apoptosis (Ma and Vosseller, 2013). Although it is likely that there are more cancer‐related OGPs involved in these processes, many remain unidentified. In this study, we used Click‐iT™ O‐GlcNAc Enzymatic Labeling System and mass spectrometry to reveal OGPs that are related to the progression of CCA. Among these, hnRNP‐K was shown to be O‐GlcNAcylated and associated with malignant progression phenotypes of CCA cells. In addition, this study is the first demonstration that O‐GlcNAc modification has an impact on nuclear translocation of hnRNP‐K and mediates the migratory ability of CCA cells. The association of O‐GlcNAcylation and the nuclear translocation of hnRNP‐K with metastasis and poor patient outcome were also demonstrated.

The contribution of O‐GlcNAcylation in the progression of CCA has been sequentially reported (Phoomak *et al*., 2012, 2016, 2017). Immunohistochemistry of OGP, OGT, and OGA in tumor tissues from patients revealed that CCA tissues had increased expression of OGPs which resulted from the increase of OGT and decrease of OGA expression. Correlation of high OGPs in CCA tissues with poor clinical outcomes of CCA patients was observed (Phoomak *et al*., 2012). Recently, O‐GlcNAcylation was shown to enhance progressive phenotypes of CCA cells by increasing high mannose N‐linked glycans at the cell surface through regulation of FOXO3 and MAN1A1 expression (Phoomak *et al*., 2018). The connection of O‐GlcNAcylation to the migration and invasion abilities of CCA cells was shown to be partly via activation of nuclear translocation of NF‐κB (Phoomak *et al*., 2016). Reducing the cellular O‐GlcNAcylation by siOGT, however, suppressed migration and invasion abilities of CCA cells to a lower extent than the inactivation of NF‐κB (Phoomak *et al*., 2016). This implies that there might be other O‐GlcNAcylated proteins together with NF‐κB that modulate progression of CCA cells. In the present study, novel OGPs that associated with progressive phenotypes of CCA were explored.

O‐GlcNAc is particularly difficult to detect due to biological and technical challenges. First, cells contain high levels of hydrolase enzymes which can rapidly remove O‐GlcNAc when cells are damaged or lysed, resulting in loss of O‐GlcNAc during protein isolation (Greis and Hart, 1998; Hart *et al*., 2007). Second, O‐GlcNAc appears on a protein at substoichiometric amounts and easily falls off when it is ionized in a mass spectrometer (Greis and Hart, 1998). Third, the signal of O‐GlcNAcylated peptides, if remained, is almost always suppressed by the higher abundance of unmodified peptides (Greis and Hart, 1998). To determine the OGPs that modulate progression of CCA cells, we first increased the signal of OGPs by inhibiting the activity of OGA (an enzyme that removes GlcNAc from the proteins) with PUGNAc. The treatment did increase OGP levels in both CCA cell lines (Fig. 1A) and enhanced the progressive phenotypes of CCA cells. These results support the association of O‐GlcNAcylation and progression of CCA cells. The sensitivity to detect O‐GlcNAcylated peptides was elevated using Click‐iT™ *O*‐GlcNAc Enzymatic Labeling System, which stabilized the GlcNAc moieties on the peptide by GalNAz labeling. The system allowed us to select and detect only O‐GlcNAcylated peptides for mass spectrometric analysis. In this study, there were over 100 OGPs detected, of which 21 were commonly found in both CCA cell lines, KKU‐213 and KKU‐214 (Table 1).

The OGPs found in this study were checked against a curated database of experimentally verified O‐GlcNAcylated proteins using the Database of O‐GlcNAcylated Proteins and Sites (dbOGAP) (Wang *et al*., 2011). Twelve proteins were identified as novel OGPs. Among these, hnRNP‐K, a member of the RNA/DNA‐binding protein family, was selected for further verification (Lu and Gao, 2016). hnRNP‐K has a unique RNA‐ and DNA‐binding component of ribonucleoproteins, which is involved in several cellular processes, including chromatin remodeling, transcription, mRNA processing, translation, nuclear transport, signal transduction, and DNA repair (Gao *et al*., 2013; Lu and Gao, 2016). It can be further modified by several post‐translational modifications, including phosphorylation, that regulates its function and interactions with different binding partners (Barboro *et al*., 2014a). There are several studies that have indicated the significant roles of hnRNP‐K in the development and progression of several cancers, including cancers of the bladder (Chen *et al*., 2017), breast (Dhanjal *et al*., 2014), colon (Zhang *et al*., 2016b), pancreas (Zhou *et al*., 2010), prostate (Barboro *et al*., 2014b), lung (Li *et al*., 2011), cervix (Zhang *et al*., 2016a), and liver (Xiao *et al*., 2013).

In the present study, we demonstrated that hnRNP‐K expression is related to cell proliferation, migration, and invasion which are hallmarks of cancer progression. Silencing of hnRNP‐K expression with specific siRNA significantly decreased cell growth, migration, and invasion of both CCA cell lines tested. Suppression of hnRNP‐K expression decreased the key effectors of cell growth (cyclin D1 and XIAP) and increased the level of cleaved caspase 3, a marker of apoptosis (Fig. 4A,B). The impact of hnRNP‐K on cell proliferation was firstly shown in colon (Sugimasa *et al*., 2015), liver, and bladder cancers (Chen *et al*., 2017; Xiao *et al*., 2013). In the current study, hnRNP‐K was shown to be involved in cell migration and invasion of CCA cells. Reduced hnRNP‐K expression significantly diminished the migration and invasion abilities of CCA cells and decreased the expression of the effector markers of migration and invasion—EMT markers (cadherin, claudin‐1, vimentin, slug) and metastasis‐related proteins (MMP2, MMP7). The association of these markers and progressive phenotypes has been reported in several cancer cells (Chung *et al*., 2014; Gao *et al*., 2013; Zhang *et al*., 2016b; Zhou *et al*., 2010). Cyclin D1 and MMP2 have been demonstrated to be the direct downstream targets of hnRNP‐K. Decreased expression of cyclin D1 was shown in hnRNP‐K suppressing bladder cancer cells (Chen *et al*., 2017). In addition, increased transcription activity and mRNA level of MMP2 were shown in hnRNP‐K enhancing colorectal cancer cell lines (Zhu *et al*., 2017). Whether the EMT markers (cadherin, claudin‐1, vimentin, slug) and MMP7 are direct downstream targets of hnRNP‐K remain to be explored.

The connection of O‐GlcNAcylation, hnRNP‐K, and progression of CCA cells was further supported by the study of KKU‐100 which exhibited lower migration and invasion activities than KKU‐213. The levels of O‐GlcNAcylation and hnRNP‐K expression as well as the downstream signals of cell proliferation and EMT markers related to hnRNP‐K were also lower in KKU‐100 than those in KKU‐213. The association of hnRNP‐K and O‐GlcNAcylation with cell migration is irrespective of cell type, as monitoring of hnRNP‐K expression or O‐GlcNAcylation levels was also able to affect the migratory ability of the immortal cholangiocyte, MMNK1 and a less aggressive CCA cell line, KKU‐100 (Fig. S4). These collective results establish a correlation between the expression of hnRNP‐K and O‐GlcNAcylation with the migratory ability of CCA cells.

For this study, hnRNP‐K was justified as a novel OGP based on the analysis using Database of O‐GlcNAcylated Proteins and Sites (dbOGAP) (Wang *et al*., 2011). However, more recently, O‐GlcNAcylation of hnRNP‐K has been identified and reported previously in breast cancer (Champattanachai *et al*., 2013; Drougat *et al*., 2012). The modulation of O‐GlcNAc on hnRNP‐K was confirmed by anti‐OGP and anti‐hnRNP‐K immunoprecipitation as well as sWGA pull‐down assays (Fig. 2). In agreement with this phenomenon, the elevation of O‐GlcNAcylation by PUGNAc treatment also increased the level of O‐GlcNAcylated hnRNP‐K in CCA cells. This evidence provides a link between global O‐GlcNAcylation and O‐GlcNAcylated hnRNP‐K.

Even though the modification of hnRNP‐K by O‐GlcNAcylation has been shown, the effect of O‐GlcNAcylation on the regulation of hnRNP‐K expression and action is unknown. The present study reported for the first time the effect of GlcNAc modification on the nuclear translocation of hnRNP‐K. The O‐GlcNAcylation of CCA cells was modulated using siOGT or PUGNAc treatment. Treated cells with siOGT significantly decreased O‐GlcNAcylation levels whereas PUGNAc treatment reversed the observation (Fig. 5A). Modulating levels of O‐GlcNAcylation have no effect on the expression of hnRNP‐K but did affect the O‐GlcNAcylated level of hnRNP‐K (Fig. 2). As hnRNP‐K action is in the nucleus, we then investigated the effect of O‐GlcNAcylation on nuclear translocation of hnRNP‐K. To visualize nuclear hnRNP‐K in relation with O‐GlcNAcylation, the immunocytofluorescence of hnRNP‐K was assessed in CCA cells treated with scramble siRNA or siOGT. As demonstrated in Fig. 5B, suppression of O‐GlcNAcylation by siOGT in KKU‐213 and KKU‐214 cells significantly reduced the number of cells with positive nuclear hnRNP‐K. Conversely, increased O‐GlcNAcylation in KKU‐100 by PUGNAc treatment increased the number of cells with nuclear hnRNP‐K (Fig. S5C). O‐GlcNAc‐induced nuclear translocation of other proteins besides hnRNP‐K has also been observed, for example, NF‐kB in CCA (Phoomak *et al*., 2016) and lung cancer (Yang *et al*., 2008); hnRNP‐A1 (Roth and Khalaila, 2017) and β‐catenin in colorectal cancer (Olivier‐Van Stichelen *et al*., 2012).

The significance of nuclear localization of hnRNP‐K was linked to migration of CCA cells by the observation that almost all migrated cells of siOGT‐treated cells had nuclear hnRNP‐K. The positive associations of O‐GlcNAcylation and nuclear hnRNP‐K as well as progressive phenotypes were also evident in tumor tissues from CCA patients (Fig. 6A–D). High level of nuclear hnRNP‐K in CCA tissues was associated with metastatic stage and shorter survival of CCA patients. The association of hnRNP‐K with poor prognosis has also been reported in colon cancer (Carpenter *et al*., 2006).

Collectively, the results above demonstrated the function of O‐GlcNAcylation on nuclear translocation of hnRNP‐K. Whether this association is the direct effect of O‐GlcNAcylation on hnRNP‐K, however, is still obscure. It has been shown that nuclear translocation of hnRNP‐K is mediated via activation of Akt (Barboro *et al*., 2014b; Li *et al*., 2011), which is also regulated by O‐GlcNAcylation (Phoomak *et al*., 2016). In CCA cells, the association of nuclear translocation of hnRNP‐K and O‐GlcNAcylation is possibly the direct effect of O‐GlcNAcylation on hnRNP‐K or formed indirectly via Akt activation. Further experiments using site‐directed mutagenesis of O‐GlcNAcylation on hnRNP‐K are required for the complete understanding of the precise role of O‐GlcNAcylation on nuclear translocation of hnRNP‐K.

Our results also underscore the impact of nuclear translocation of hnRNP‐K on the migratory ability of CCA cells. First, siOGT treatment inhibited nuclear translocation of hnRNP‐K and concurrently decreased migration of CCA cells. Second, almost all migrated cells detected in migration assay had positive nuclear hnRNP‐K. These findings prompt further development of inhibitors of hnRNP‐K nuclear translocation to diminish CCA progression.

## 5 Conclusion

Primarily in the nucleus and cytoplasm, 12 novel OGPs associated with progression of cancer were revealed in CCA cells. Of these, hnRNP‐K was validated for its O‐GlcNAc modification and its molecular mechanism in promoting progression of CCA (Fig. 6E). The impact of hnRNP‐K on progressive phenotypes—cell growth, migration, and invasion—was emphasized. O‐GlcNAcylation was proved to be necessary for nuclear translocation of hnRNP‐K that subsequently activates several downstream targets of hnRNP‐K (cyclin D1, XIAP, caspase 3, EMT markers, and MMP2 and MMP7). Expression of nuclear hnRNP‐K in tumor tissues predicted the metastatic stage and associated with poor patient outcome. Inhibition of nuclear translocation of hnRNP‐K may be a new strategy for CCA treatment.

## Conflict of interest

The authors declare no conflict of interest.

## Author contributions

CP, DP, AS, KS, CBL, and SW conceived and designed experiments; CP, DP, AS, and MD performed experiments; CP and DP prepared the figures; CP, DP, AS, KS, KV, and CW analyzed data; CP, DP, CBL, and SW wrote the manuscript. All authors participated in the interpretation of the studies and reviewed the manuscript.

## Supporting information


**Table S1.** List of identified OGPs in CCA cells.

**Fig. S1.** Identification of O‐GlcNAcylated proteins using Click‐iT™O‐GlcNAc Enzymatic Labeling System and mass spectrometry.
**Fig. S2.** Predicted O‐GlcNAcylated proteins in CCA cells.
**Fig. S3.** Cell proliferation during migration and invasion assays.
**Fig. S4.** Effect of hnRNP‐K and O‐GlcNAcylation on cell migration.
**Fig. S5.** Expression and localization of hnRNP‐K in CCA cell lines.
