# Supplementary material for: O‐GlcNAc‐induced nuclear translocation of hnRNP‐K is associated with progression and metastasis of cholangiocarcinoma
Source: Mol Oncol. 2019 Jan 10;13(2):338–57. doi: 10.1002/1878-0261.12406 (PMC6360360; doi:10.1002/1878-0261.12406)
Supplement: Supplementary file 2 — Fig. S1. Identification of O‐GlcNAcylated proteins using Click‐iT™O‐GlcNAc Enzymatic Labeling System and mass spectrometry. Fig. S2. Predicted O‐GlcNAcylated proteins in CCA cells. Fig. S3. Cell proliferation during migration and invasion assays. Fig. S4. Effect of hnRNP‐K and O‐GlcNAcylation on cell migration. Fig. S5. Expression and localization of hnRNP‐K in CCA cell lines. [file MOL2-13-338-s002.docx]

**Supplementary Data**


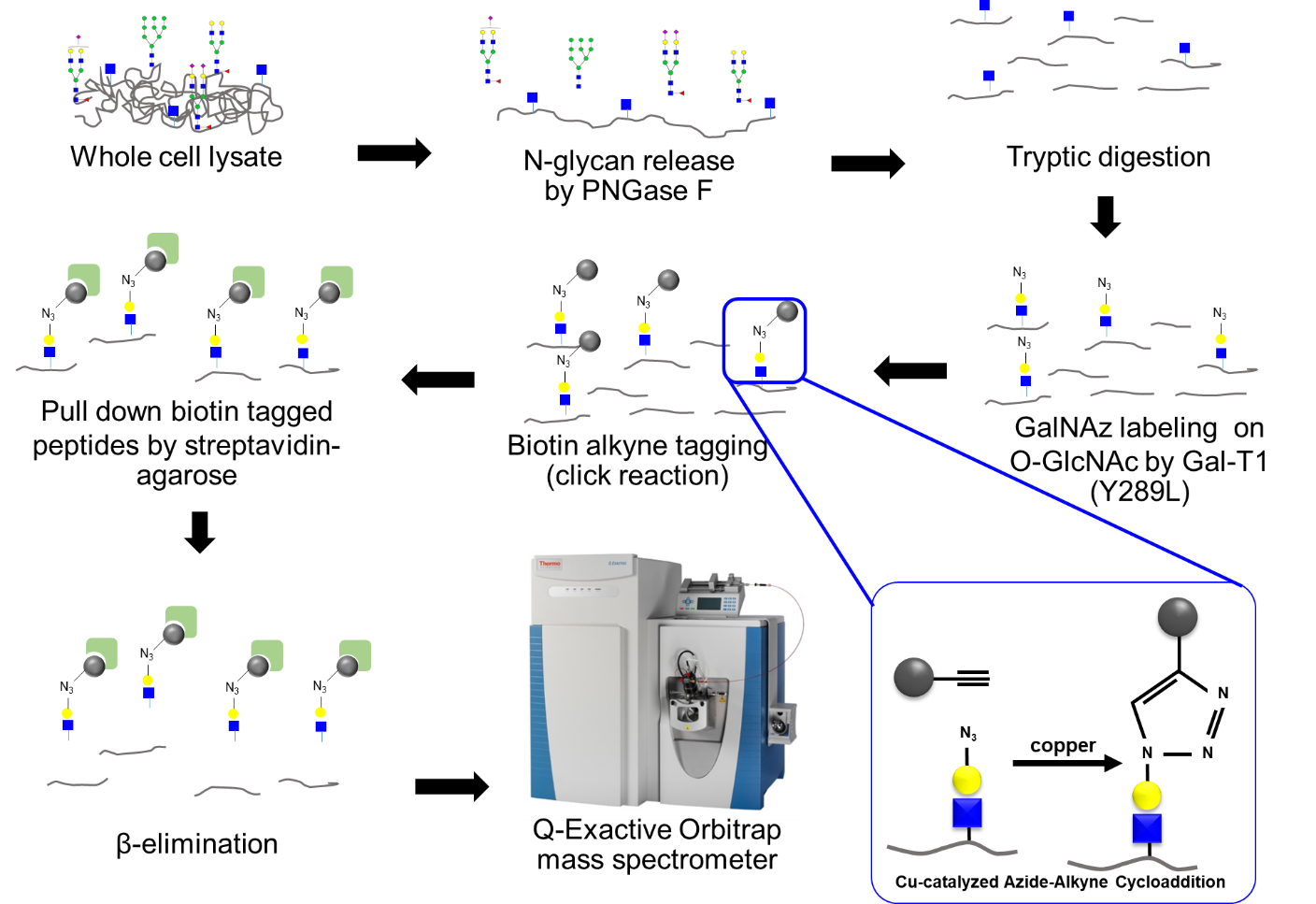


**Figure S1. Identification of O-GlcNAcylated proteins using Click-iT™ O-GlcNAc Enzymatic Labeling System and mass spectrometry.** N-glycans were firstly released by PNGase F. After tryptic digestion, the O-GlcNAc on the peptides were labelled with GalNAz and tagged with biotin via azide-alkyne cycloaddition. Biotin tagged peptides were pulled down by streptavidin-agarose and the O-GlcNAcylated peptides were released from the biotin-streptavidin complex by β-elimination. The released peptides were identified using Q-Exactive Plus Orbitrap mass spectrometry. ■ = O-GlcNAc.

**Figure S2. Predicted O-GlcNAcylated proteins in CCA cells.** A) The predicted OGPs were analyzed by The Global Proteome Machine (the GPM) and the localizations of the proteins were characterized. B) Each candidate was searched for its O-GlcNAcylated site from the O-GlcNAcylated Proteins and Sites (dbOGAP) database. Matched OGPs are grouped as “Reported”. The OGPs that were commonly found in both cell lines are displayed and ranked by intensity. C) The biological process of unreported OGPs were analyzed using PANTHER (<http://pantherdb.org/>).


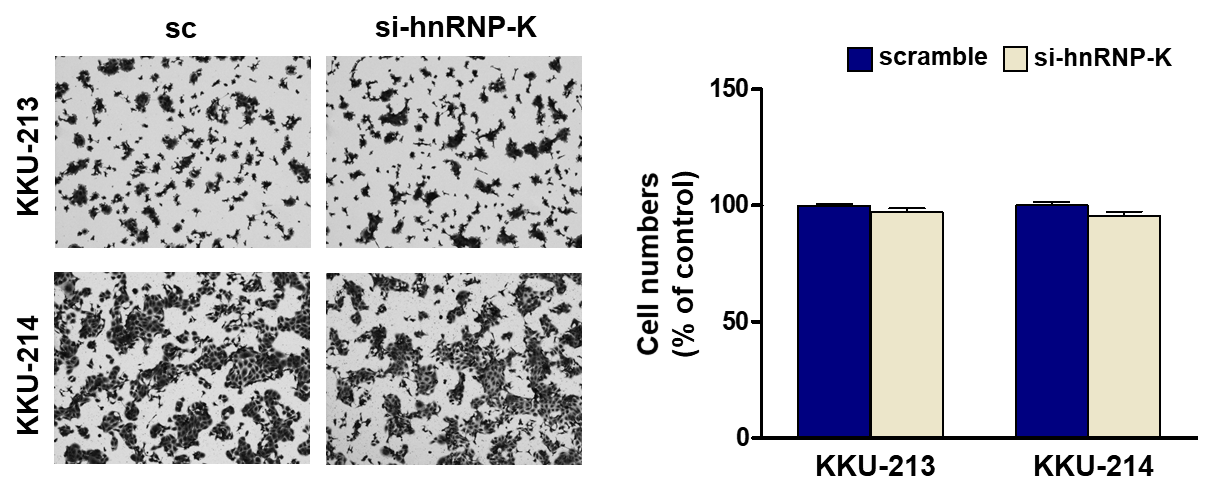


**Figure S3. Cell proliferation during migration and invasion assays.** In parallel with migration and invasion experiments, 4 x 10^4^ cells were seeded onto a 24 well plate and number of cells were determined using sulforhodamine B stain at the same time period for migration/invasion assays, 9 h for KKU-213 and 24 h for KKU-214. Cell numbers were determined and calculated as % of control. The data are mean ± SEM.


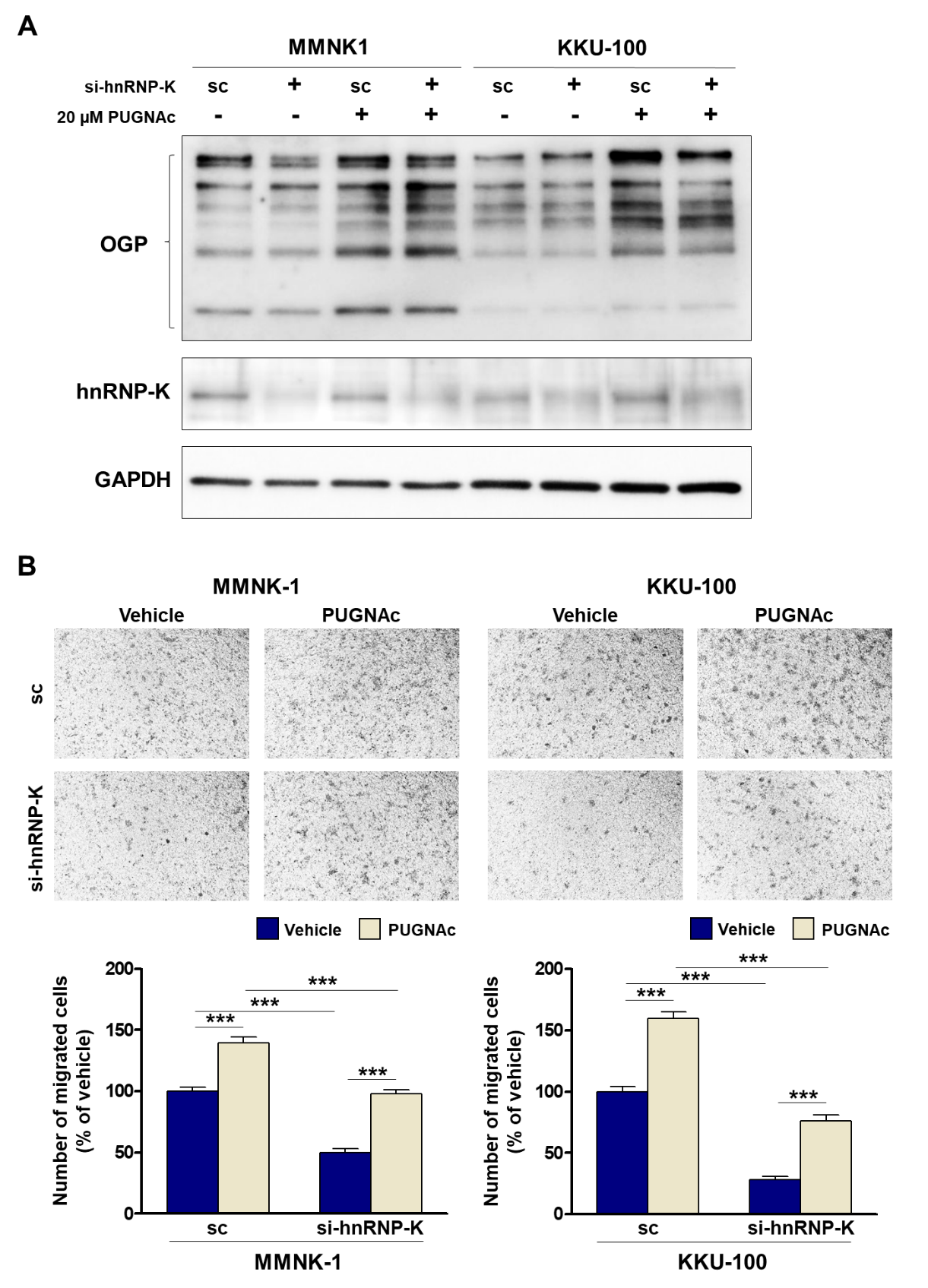


**Figure S4. Effect of hnRNP-K and O-GlcNAcylation on cell migration.** Immortalized cholangiocyte cell line, MMNK1, and CCA cell line, KKU-100, were treated with scramble siRNA (sc) or si-hnRNP-K in combination with (+)/without (-) 20 µM PUGNAc, an OGA inhibitor, for 24 h. A) The levels of OGP and hnRNP-K were determined using western blot analysis. B) The migratory ability of the cells was compared using Boyden’s chamber assay. The results represent one of two independent experiments (Mean ± SEM, ****P* < 0.001, Student’s t-test).


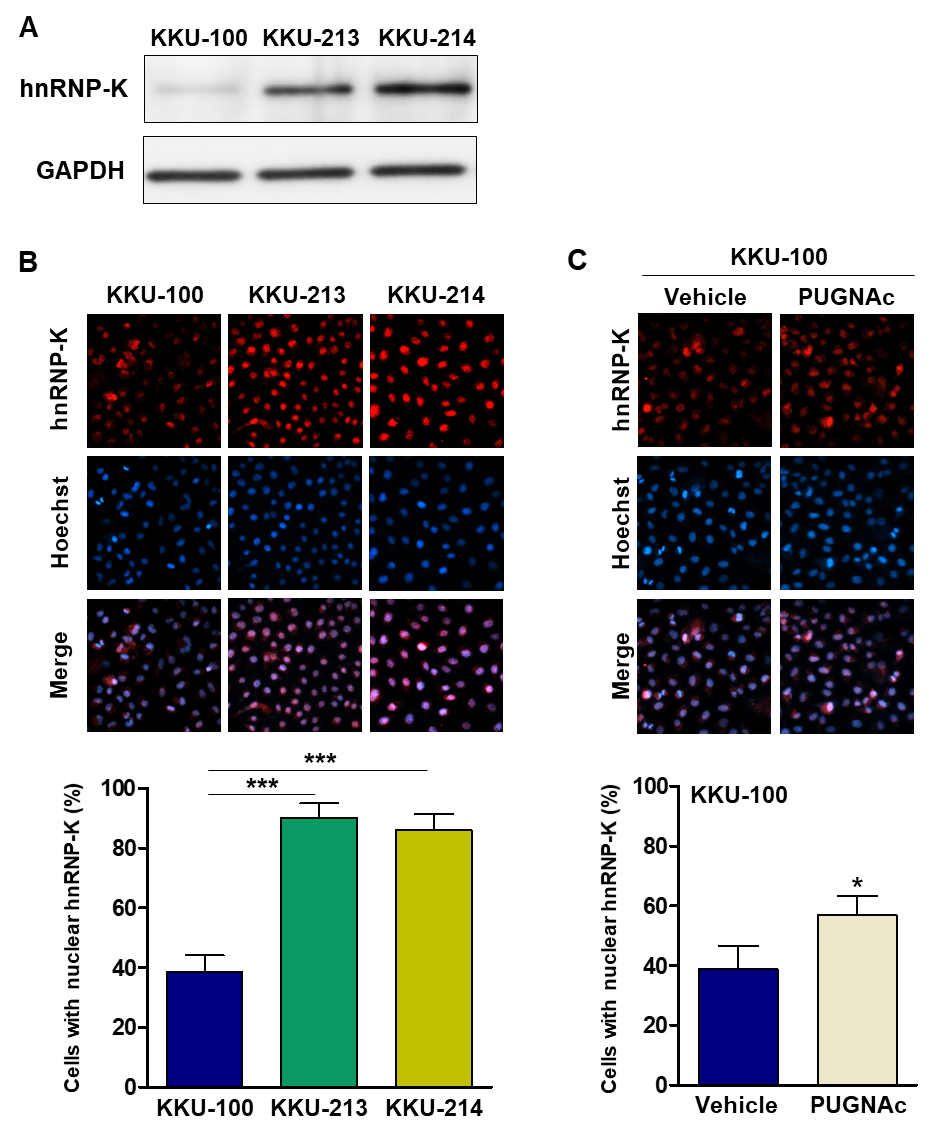


**Figure S5. Expression and localization of hnRNP-K in CCA cell lines.** Immunocytofluorescent staining of hnRNP-K was performed in KKU-100 comparatively with KKU-213 and KKU-214 cell lines. Number of cells with nuclear hnRNP-K were counted. A) Western blot analysis of hnRNP-K expression. B) Nuclear localization of hnRNP-K was determined using immunofluorescent staining. C) KKU-100 cells were treated with/without PUGNAc and localization of hnRNP-K was determined. The data are mean ± SD. Astericks indicate statistical significance, **P* < 0.05, ****P* < 0.001 (Student’s t-test).
